# Influence of Xanthan Gum Addition on the Short- and Long-Term Retrogradation of Corn Starches of Various Amylose Content

**DOI:** 10.3390/polym14030452

**Published:** 2022-01-23

**Authors:** Magdalena Krystyjan, Anna Dobosz-Kobędza, Marek Sikora, Hanna Maria Baranowska

**Affiliations:** 1 Department of Carbohydrates Technology and Cereal Processing, Faculty of Food Technology, University of Agriculture in Krakow, Balicka Street 122, 30-149 Krakow, Poland; anna.dobosz88@gmail.com (A.D.-K.); rrsikora@cyf-kr.edu.pl (M.S.); 2 Department of Physics and Biophysics, Poznan University of Life Sciences, 38/42 Wojska Polskiego Street, 60-637 Poznan, Poland; hanna.baranowska@up.poznan.pl

**Keywords:** retrogradation, corn starch, xanthan gum, gels, viscoelasticity, syneresis, storage stability

## Abstract

Starch retrogradation is a complex process and in most food products is undesirable. Knowing and understanding the mechanisms and factors that influence this process may become the key to a better and innovative approach to food design. In this paper, we investigated the effect of 0%, 0.05% and 0.20% (w/w) xanthan gum (XG) addition on the short- and long-term retrogradation of 4%, 5% and 6% corn starch gels, depending on the amylose/amylopectin ratio in the starch. The changes were monitored throughout 90 days. The pasting characteristics of blends, rheological and texture analyses, as well as syneresis, revealed that XG stabilizes the starch in the short term, but it does not inhibit retrogradation caused by amylopectin. After 30 days of storage, the destabilization of the starch-hydrocolloid mixture was observed. Based on the obtained results, a probable mechanism for the retrogradation of corn starch process in the presence of xanthan gum was proposed.

## 1. Introduction

Retrogradation of starch is a complex process which depends on many factors. The amylose (AM) to amylopectin (AP) ratio seems to have dominant role [1]. The greater the amylose content in the starch, the more it is susceptible to retrogradation process [2]. The amount of lipid complex, amylose chains and phosphorus content also affect the rate of retrogradation of amylose. In the case of amylopectin, the unit chain length and extent of branching seem to be of importance [3]. Therefore, this process can be considered in two stages. In the first one, called short-term retrogradation, the dominant role is played by amylose, which forms double-helical associations of 40–70 glucose units [4], whereas the second phase of retrogradation, called long-term, results from the recrystallisation of amylopectin [5,6] by reassociation of the outermost short branches [1]. In addition, the rate of retrogradation is also influenced by such factors as the ratio of water to starch, storage temperature and presence of nonstarch components [7].

Hydrocolloids used as thickeners, gelling agents and/or stabilizing starch systems may slow down/inhibit or, on the contrary, accelerate the process of starch retrogradation [8,9]. However, due to their different origins, and hence their different structure, it was not possible to propose a single common mechanism that could explain the way they inhibit/accelerate starch retrogradation. Fu and BeMiller [6] describe possible cases of starch–hydrocolloids interactions. In the first one, the addition of a hydrocolloid to starch results in the immobilization of water molecules, and thus the effective concentration of polysaccharide molecules in the continuous phase increases. A phase separation is also possible, due to thermodynamic incompatibility of polysaccharide molecules, which, in a further stage, leads to the mutual exclusion of each composite. This is also confirmed by other researchers [10,11,12]. A third possibility is the interaction between polymer molecules [6]. The starch retrogradation process has been studied for a long time.

However, for technical, technological and analytical reasons, there were numerous limitations that prevented an in-depth analysis of this issue. In the year of 2000, Karim et al. [13] described the latest methods for the study of starch retrogradation. The multitude of factors determining the retrogradation phenomenon is so large that research in this area is carried out to this day. One of the important factors controlling this process is the presence of thickeners other than starch in the solution, i.e., nonstarch hydrocolloids. The most commonly used polysaccharide hydrocolloids in the food industry include, among others, carrageenans, galactomannans and xanthan gum. The influence of the latter on the retrogradation of starch has been studied by many authors, but the changes taking place in starch gels were monitored for no longer than 60 days of storage [6,14]. Since food products based on polysaccharide hydrocolloids very often have a longer shelf life than 60 days, it seems necessary to determine changes in the structure of starch gels over a longer period of storage. There are no products based on corn starch gels that could be stored for such a long time (90 days) without unfavorable changes in the structure. Therefore, in order to be able to prevent or limit these adverse properties of starch, it is necessary to understand the mechanism and factors that govern them. This will allow for the effective manipulation of production parameters in the future in order to obtain products based on or with the addition of starch gels with stable structure. Therefore, the aim of this study was to determine the influence of xanthan gum addition on short- and long-term retrogradation of corn starch monitored these changes across 90 days. Furthermore, the goal of the study was to propose, based on the obtained results, a probable mechanism for the retrogradation of starch process in the presence of xanthan gum. In 2019, we started to study changes in starch gels, upon 90 days of storage, including plain potato starch gels and mixtures with various hydrocolloids [15,16]. We also have been conducting research on retrogradation of corn starch for 90 days [17,18]. The work is a continuation of the research we have started in this area. Its purpose is to determine changes, not only in the short-term, but also in the long-term retrogradation of corn starch stored for 90 days and in the presence of xanthan gum. So far, no research has been conducted on this phenomenon throughout such a long period of time. An attempt was also made to answer the question of how xanthan gum contributes to the retrogradation process of corn starch, especially long-term (up to 90 days), and to indicate a potential governing mechanism.

## 2. Materials and Methods

### 2.1. Materials

Normal corn starches denoted as NCS1 (commercial C*Gel 03401, LOT number: 0137610) and NCS2 (commercial C*Gel 03401, LOT number: 03317825) were purchased from Cargill (Warsaw, Poland). Waxy corn starch (WCS) (commercial C*Gel 04201, LOT number: 01344917) was purchased from Cargill (Warsaw, Poland) and contained 0.75% amylose. The amylose contents of starch were presented in our previous paper, Sikora et al. 2018 [17], and are respectively NCS1—21.61%, NCS2—20.48% and WCS—0.75%.

Xanthan gum (XG) from *Xanthomonas campestris* (G1253-500G, Lot#100M0218V) was purchased from Sigma–Aldrich (St. Louis, MO, USA).

### 2.2. Sample Preparation

The samples for rheological and textural measurements, as well as for syneresis, were prepared and measured according to Sikora et al. [17] in which appropriate amounts of starch and xanthan gum were mixed together in water solution. The 0.4% aqueous solution of sodium azide was added to protect samples from microorganisms during the storage. Samples were stirred mechanically at 400 rpm firstly for 10 min at 25 ± 2 °C and then for 30 min at 95 ± 2 °C. The concentration of starch in pastes and gels was 4%, 5% and 6% (w/w, dry basis) (they were control samples). The combinations of binary polysaccharides mixtures (starch + XG) were 4%, 5% and 6% (w/w, dry basis) for starch and 0.05% and 0.20% (w/w, dry matter) for xanthan gum.

### 2.3. Pasting Characteristics

Analysis was conducted according to our previous method [19] by the use of a Micro Visco-Amylo-Graph (Brabender, Duisburg, Germany) viscometer. Samples containing 4%, 5% and 6% (w/w) of starch and 0%, 0.05% and 0.20% of XG were pasted following the subsequent regime: (1) increase in temperature: 4.5 °C/min, (2) temperature profile: 30 °C–96 °C–50 °C, (3) maintaining temperature of 96 °C for 10 min and 50 °C for 1 min and (4) rotor velocity: 250 rpm. Measurements were duplicated.

### 2.4. Dynamic Viscoelasticity

Freshly prepared pastes were transferred into disposable aluminum foils placed in nonventilated polystyrene Petri dishes in the amount of 4 cm^3^. They were left at RT for 2 h then placed in a refrigerator at 6 °C. Oscillation measurements were performed with a RheoStress RS 6000 (Thermo Scientific, Karlsruhe, Germany) rotary rheometer equipped with a plate–plate P35 Ti measuring system (35 mm diameter) and temperature control module based on the Peltier system. In order to adjust the gap, the distance between them was reduced with the 0.15 mm/s rate until the normal force reached 0.2 N. The estimations were performed after the samples were prepared (after 1 and 2 h) and then after 1, 2, 10, 30, 60 and 90 days storage. The oscillation measurements included running mechanical spectra in the range of 0.1 to 10 Hz at 25 °C and 0.3% deformation fitting the range of linear viscoelasticity. The estimations were run in duplicates.

### 2.5. Gel Hardness

The measurements for the gels were performed according to Krystyjan et al. [5] procedure with some modifications. After preparation (according to the Section 2.2), samples (45 cm^3^) were kept in polypropylene containers (inner diameter—39 mm, height—63 mm) firstly at room temperature for 2 h and then in refrigerated at 6 °C. Measurements of the hardness of gels with the Texture Analyzer TA.XT plus (Stable MicroSystems Ltd., Godalming, UK) were performed for freshly prepared samples (1 and 2 h) and after their storing for 1, 2, 10, 30, 60 and 90 days. The penetration test involved P/0.5 cylinder taken as a measurement probe. It was immersed 10 mm deep, and its velocity was 1.0 mm/s. The measurements were run in triplicates.

### 2.6. Syneresis

The samples were prepared in the same way as for textural analysis (Section 2.5) and kept refrigerated at 6 °C. Syneresis was determined as a difference between the original weights of the sample (after preparation) and after removing water from it. The water was removed using a polyethylene Pasteur pipette and then by drying surfaces of the samples and containers with a tissue. The estimations were performed in duplicates. The water loss (in %) was calculated from Equation (1):

(1) $$X=\frac{b-c}{b-a}\cdot 100$$

where:

*a*—the weight of empty container with its cap (g),
*b*—the weight of container with its cap after filling it with a sample (g),
*c*—the weight of container with its cap and sample after the separated water was removed (g).

### 2.7. Statistics

Statistical analysis involved a Statistica 12.5 (StatSoft, Tulsa, OK, USA) software employing mono- and bifactorial analysis of variance and the Duncan’s test for checking the significance of the differences at α = 0.05.

## 3. Results and Discussion

### 3.1. Pasting Characteristic of Blends

Table 1, Table 2 and Table 3 present the parameters of the pasting characteristics of native and waxy maize starches and their mixtures with xanthan gum. The starch pasting process started at different temperatures, depending on the type of starch used. Among the studied corn starches, waxy starch with the lowest amylose content was characterized by the lowest temperature needed to start the pasting process, and the difference was from 7.1 to 16.5° C compared to their native counterparts (NCS1 and NCS2) (Table 1, Table 2 and Table 3).

The presence of hydrocolloid in the mixture affected the starch pasting. A significant decrease in the initial pasting temperature and an increase in the viscosity of the binary systems were noticed. A similar tendency was observed by Mandala and Bayas [20], who studied the influence of xanthan gum on the rheological properties of wheat starch. Using two different blending techniques, they found that heated aqueous starch solution in the presence of gum led to an exclusion effect, which consisted in limiting access to water for starch granules by wrapping them with a thin film of gum, which, in turn, caused point increases in concentration of hydrocolloid in the continuous phase. In this case, phase separation was inevitable [21]. A similar assumption was made by Baranowska et al. [9], who found that the pasting and gelling process of starch depended on the availability of water molecules, and this property, in turn, was shaped by factors such as: the ability of hydrocolloids to retain water molecules, conformational changes of polysaccharides and inhibition of gelation, which was supposed to be the result of an interaction between hydrocolloids and starch granules.

Table 1, Table 2 and Table 3 present the setback parameter, which is the difference between the viscosity after cooling the paste to 50 °C and the minimum viscosity and which is helpful in determining the tendency of starch to retrogradation [22]. It is assumed that the higher the setback value, the greater the retrogradation tendency of pure starch system [22]. Analyzing the results presented in Table 1, Table 2 and Table 3, it can be concluded that the setback values of starch pastes increased with the increase in the concentration, as well as the amylose content in the starch. The waxy starch at concentration of 4% had the lowest setback parameter, and this value was even 30% lower than that for native starch (conc. 4%) (NCS2) (Table 1, Table 2 and Table 3). At a higher concentration of starch (5% and 6%), the effect was more pronounced. Therefore, it can be concluded that the amylose content is an important parameter in controlling susceptibility to retrogradation on cooling, which is in agreement with the results of pasting characteristics obtained by Krystyjan et al. [5] and Dobosz et al. [23].

The presence of the hydrocolloid in the mixture influenced the starch pasting characteristics and the setback parameter. XG decreased the value of the setback of native corn starch pastes in each concentration of the starch (Table 1 and Table 2), which may suggest that, in two-component systems, the starch retrogradation was lower. In the case of waxy starch, slight changes in the values of this parameter were noticed (Table 3). Literature data confirm that amylose with low molecular weight retrogrades the fastest [5,23,24], because it is more mobile and therefore more likely to organize in solution [24]. In turn, amylopectin molecules with small and medium length of the outermost terminal branches of the chains, which are involved in the formation of crystals during retrogradation, retrograde more slowly compared to those of longer length [5,25]. However, there is a minimum chain length required for both amylose and amylopectin, at which the phenomenon of their aggregation takes place. In the case of amylose, it is 10 anhydroglucose units, which corresponds to the length of the maltooligosaccharides [26]. According to Gudmundsson [26], short-chain amylose with a degree of polymerization of 8 does not show crystallization. Amylopectin chains shorter than 15 anhydroglucose residues do not take part in crystallization [26,27]. Thus, native starches, with a high content of amylose, retrograde faster and to a greater extent than waxy starches that have little or no amylose. Therefore, short-term starch retrogradation is attributed to amylose, whereas the changes occurring in the later period are caused by amylopectin [5]. Based on the setback parameter (Table 3), it can be concluded that WCS will show a lower tendency to retrogradation than its native counterparts. The xanthan gum added to the system may reduce the short-term retrogradation, which confirmed Krystyjan et al. [5], He et al. [28] and Hong et al. [29]. Pongsawatmanit et al. [30], however, made other observations. According to them, xanthan gum promoted short-term retrogradation process. In both cases, the results obtained may be as true as possible because, as Fu and BeMiller [6] explained, the reasons for this are the differences in the method of sample preparation and measurement techniques. The authors give an example in which starch pastes prepared using low and high shear contain different amounts of swollen granules and/or granule fragments in mixture.

### 3.2. Rheology of Gels

**Figure 1:**
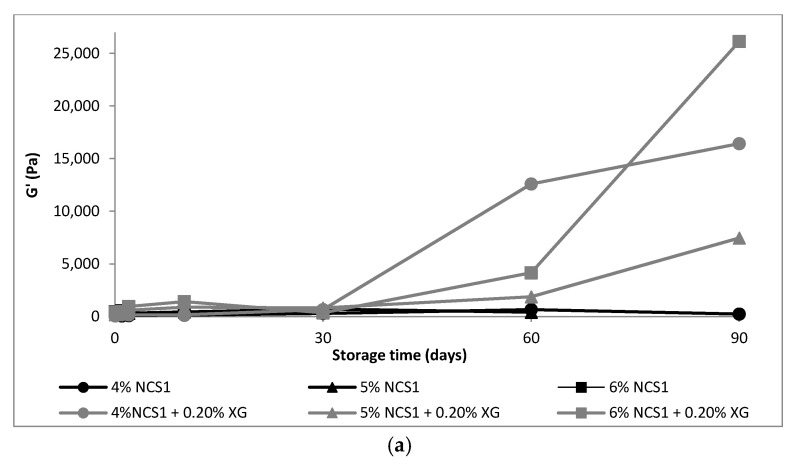

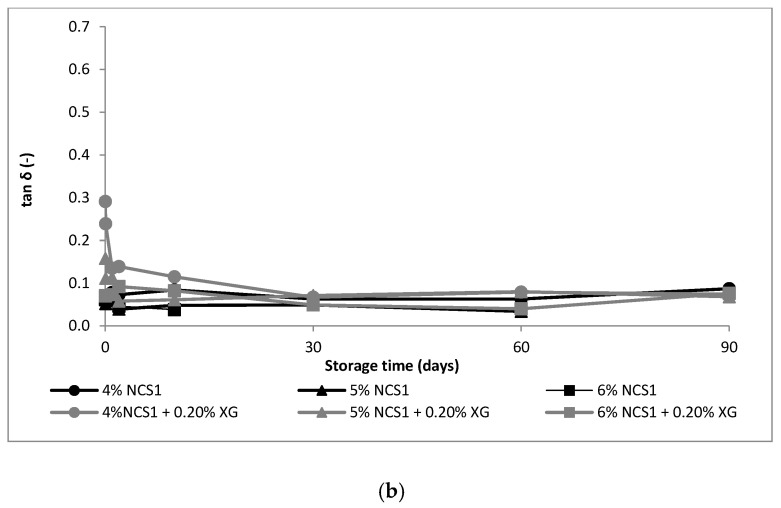
Mechanical parameters of 4–6% NCS1 gels without and with 0.2% XG: (**a**) *G*′, (**b**) *tan* *δ* = (*G*″/*G*′) measured at 1 Hz. NCS1—normal corn starch with higher amylose content, NCS2—normal corn starch with lower amylose content, WCS—waxy corn starch, XG—xanthan gum. Results for NCS1, NCS2 and WCS without XG were published in our former paper Sikora et al., 2018 [17].

**Figure 2:**
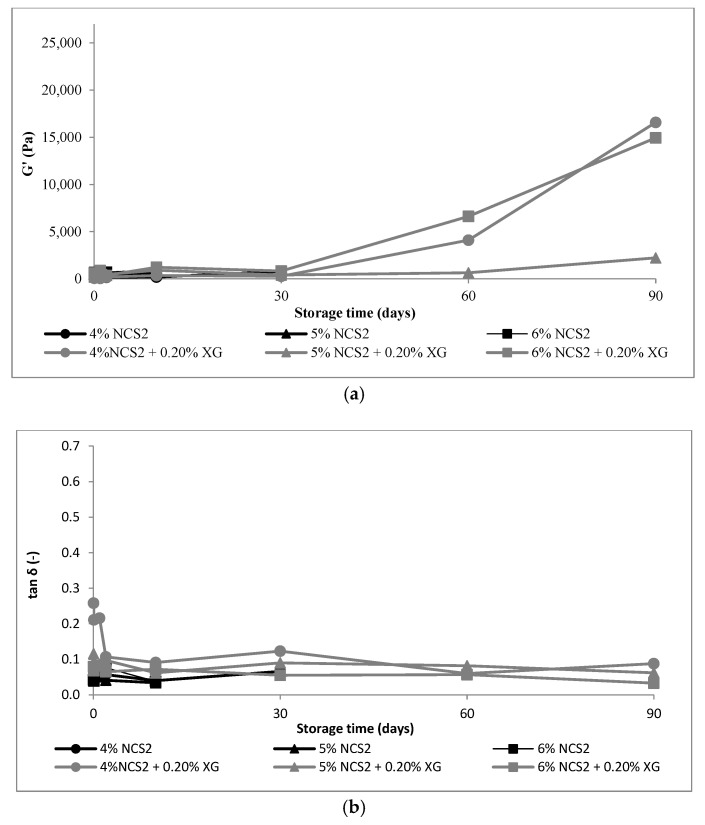
Mechanical parameters of 4–6% NCS2 gels without and with 0.2% XG: (**a**) *G*′, (**b**) *tan* *δ* = (*G*″/*G*′) measured at 1 Hz. NCS1—normal corn starch with higher amylose content, NCS2—normal corn starch with lower amylose content, WCS—waxy corn starch, XG—xanthan gum. Results for NCS1, NCS2 and WCS without XG were published in our former paper Sikora et al., 2018 [17].

**Figure 3:**
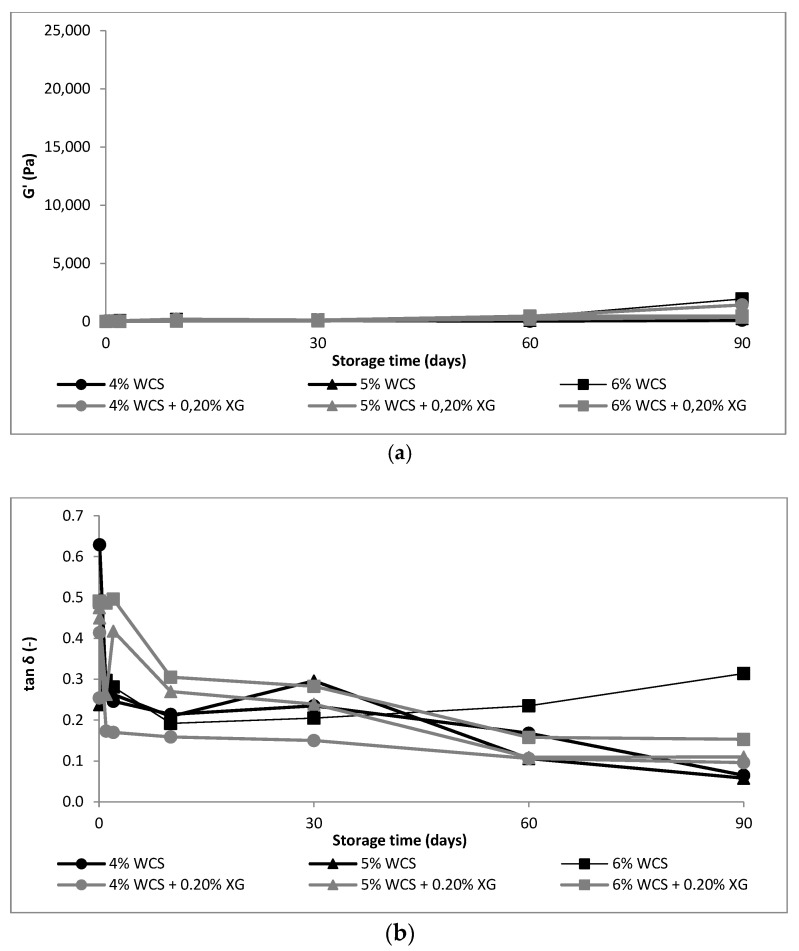
Mechanical parameters of 4–6% WCS gels without and with 0.2% XG: (**a**) *G*′, (**b**) *tan* *δ* = (*G*″/*G*′) measured at 1 Hz. NCS1—normal corn starch with higher amylose content, NCS2—normal corn starch with lower amylose content, WCS—waxy corn starch, XG—xanthan gum. Results for NCS1, NCS2 and WCS without XG were published in our former paper Sikora et al., 2018 [17].

The rheological properties of starch gels without and with the addition of xanthan gum stored for 90 days are shown in Figure 1, Figure 2 and Figure 3.

A much faster increase in *G*′ modulus over time for native starches (Figure 1a and Figure 2a) than for waxy starch (Figure 3a) was observed. The storage modulus *G*′ defines the elastic properties of the tested material and, according to Li et al. [31], result from junction zones of the three-dimension network structure of the amylose and swollen granule in the system. The increase of this parameter corresponds to formation of three-dimensional network and crosslinking of starch [28,31], and it is a practical way to monitor short-term retrogradation [5,14,31]. The faster G′ increases in samples, the more intense is the retrogradation process. The *G*′ values of all the starches showed a gradual increase of *G*′ over time until 90 days (Figure 1a). A similar tendency was noticed by Li et al. [31]. However, they monitored only the short-term retrogradation, as they measured the viscoelastic properties of gels during 2 h. Hsu et al. [14] carried out measurements of changes in the structure of starch gels for a period of 14 days, and Krystyjan et al. [5], for 30 days. The longest, a 90-day retrogradation process, was monitored in our previous works [17,18,23]. Nevertheless, all authors agreed that in the first few hours after making the starch paste, after cooling, the aggregation of amylose took place, and the gel structure was formed. Retrogradation, in the opinion of Fu and BeMiller [6], is a complex phenomenon in which AM–AM, AP–AP and AM–AP interactions may take place, as well as crystallization. The authors noted, however, that AP–AP interactions may engage only entanglements [6].

Regardless of the starch concentration, an increased *G*′ due to the addition of xanthan gum was found (Figure 1a). A particularly intense increase in the modulus of elasticity was observed after 30 days of refrigerated storage in mixed systems of native starch with xanthan gum (Figure 1 and Figure 2). The increase in the *G*′ modulus in the first hours after the preparation of the starch gel with presence of hydrocolloid suggests acceleration of gel formation process, because, as Eidam et al. [32] claimed, the presence of xanthan gum, or more precisely its thickening effect, contributed to an increase in the viscosity of the entire system. The amylose molecule’s mobility was limited, and consequently, local association of the helical components was favorable, whereby a three-dimensional network structure stabilized by junction zones was formed. According to some authors, the addition of xanthan gum promoted association between swollen, pasted granules by one of two mechanisms [33]: via depletion flocculation or by intergranular association. Achayuthakan and Suphantharika [34] confirm the second mechanism, through phase separation. This phenomenon resulted from the hydrodynamic incompatibility of structurally dissimilar polymers molecules [6,10] and, as claimed by Gudmundsson et al. [26], promoted interactions between similar molecules, e.g., between AM and AP molecules in solution. Kim et al. [35] also pointed out that more than one mechanism occurred in complex systems, and it depended also on some conditions such as concentration and ratio of components, as well as methods of gel preparation and even measurements methods.

**Figure 4:**
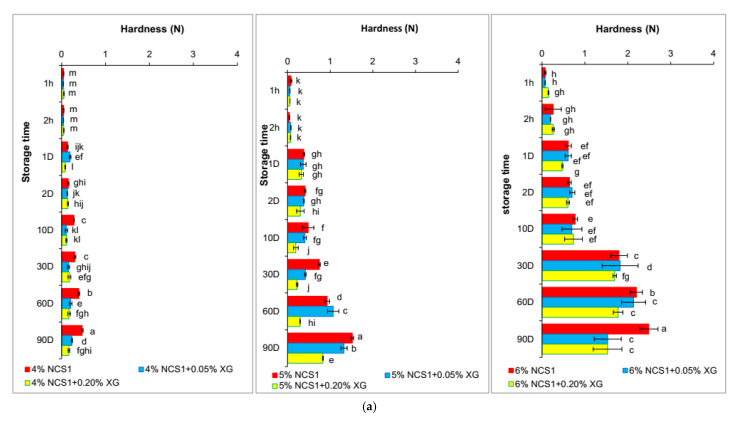

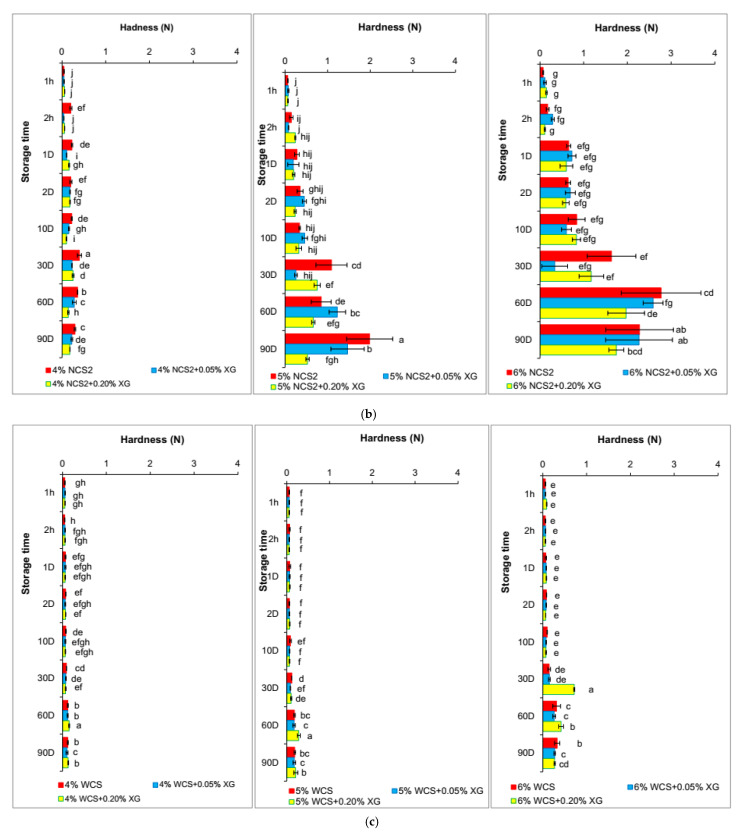
Changes in gels hardness during storage of (**a**) 4–6% NCS1 gels without and with 0.05% and 0.2% XG, (**b**) 4–6% NCS2 gels without and with 0.05% and 0.2% XG, (**c**) 4–6% WCS gels without and with 0.05% and 0.2% XG. NCS1—normal corn starch with higher amylose content, NCS2—normal corn starch with lower amylose content, WCS—waxy corn starch, XG—xanthan gum. Results for NCS1, NCS2 and WCS without XG were published in our former paper Sikora et al., 2018 [17]. Parameters denoted with the same letters do not differ statistically at the level of confidence α = 0.05.

In the case of waxy starch, the addition of XG also increased the *G*′ parameter compared to the control sample. However, that until the 30th day of storage, the effect of XG was small. On 60 and 90 days of storage, a very marked jump in the values of this modulus was observed (Figure 3). The retrogradation process of starches from different botanical sources had been proposed to be correlated with the average chain length of amylopectin [1,36]. Generally, cereal amylopectin retrogrades to a lesser extent than pea and potato amylopectin, which has been attributed to the shorter average chain lengths in the cereal amylopectin [35]. Analyzing the profile of the molecular weight distribution of corn starch (Figure 4), a clear separation into two fractions amylose and amylopectin of native corn starch (NCS1 and NCS2) was observed. On the other hand, in NCS2 starch, a clear shift of the curve profile to the left was observed, which may suggest the presence of a greater number of short and medium-length chains, whereby aggregation of short-chain amylose and amylopectin occurs in the case of the starch (NCS2) to a much greater extent or more intensely than in NCS1. It was confirmed by the increase in the value of the elastic modulus *G*′ (Figure 1 and Figure 2). The low amylose content of waxy starch influenced the molecular weight distribution profile, which showed monomodal chain length distribution [17], and it explained why the increase in *G*′ modulus over time was so small.

The addition of XG increased the value of the tan δ (*G*″/*G*′) of the mixture, which proved a greater difference between *G*′ and *G*″. The difference was more pronounced with the lower starch concentration and decreased with increasing concentration of this polysaccharide in the mixture. Thus, the influence of the gum on the rheological properties of the mixture was more pronounced at a lower starch concentration. After 30 days of storage, tan δ values decreased, and thus, the differences between *G*′ and *G*″ were smaller. This behavior may suggest that XG stabilized the system for about 30 days, and after this time, intense changes in the viscoelastic properties of the mixtures, which indicate a destabilization of the system, were observed. Thus, the effect of gum on long-term starch retrogradation was small, which may suggest no interaction between amylopectin and gum. Our assumptions were confirmed by NMR studies performed by us on the similar systems [18]. According to these results, the effect of XG was the most potent within the initial few days, and after 30 days, the effect ceased. It also was observed that molecular properties of binary WCS–XG mixture differed from the hydrocolloid-free WCS. The amount of the bulk water fraction in relation to the amount of bound water fraction was reduced, and it was manifested by longer T1 (relaxation times of spin-lattice). The water in bound fraction was more mobile, whereas the water in bulk fraction faced strong limitations. On this basis, it was concluded that XG did not interact with WCS, and the observed mobility limitations were caused by the hydrocolloid.

### 3.3. Hardness of Gels

The hardness of starch gels increased with the storage time and the content of amylose. Native starch gels (NCS1 and NCS2) showed a much faster increase in hardness during their storage than the waxy starch gels (Figure 4). In the case of the WCS, changes in this parameter were observed after the 10th day of storage, which confirms the earlier assumptions that amylopectin is responsible for the long-term retrogradation of starch [5]. However, it is worth emphasizing that even after this period the increase in hardness of the waxy starch gels was not as pronounced as in the case of native starches gels (Figure 4a,b). The high hardness of starch gels may be influenced by the higher amylose content, as well as longer amylopectin chains [37]. The progressive increase in the hardness of starch gels during their storage results, according to Miles et al. [38], comes from the ongoing retrogradation process, which in turn is related to water syneresis and crystallization of amylopectin. The presence of xanthan gum decreased the hardness of native starch gels during their storage (Figure 4a,b). In the case of waxy starch gels, the addition of this hydrocolloid had no effect on the rate of short-term retrogradation (Figure 4c). The effect of gum on the long-term retrogradation of this starch was also negligible and, in most cases, statistically insignificant. It is also worth noting that the hardness of the WCS gel with the storage time changed very little, especially for the 4% concentration of this polysaccharide. With a higher proportion of starch—6%, changes were visible, but only on the 30th, 60th and 90th days of storage. Thus, the presence of xanthan gum does not limit the long-term retrogradation of waxy starch, a conclusion that was also confirmed by Hong et al. [29], He et al. [28] and Krystyjan et al. [5].

### 3.4. Syneresis of Gels

**Figure 5:**
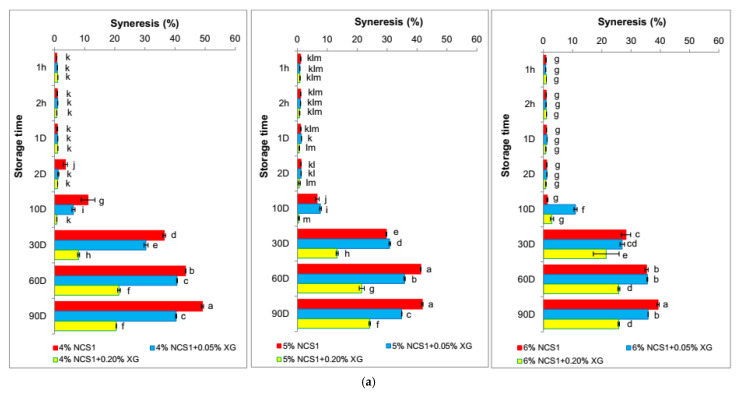

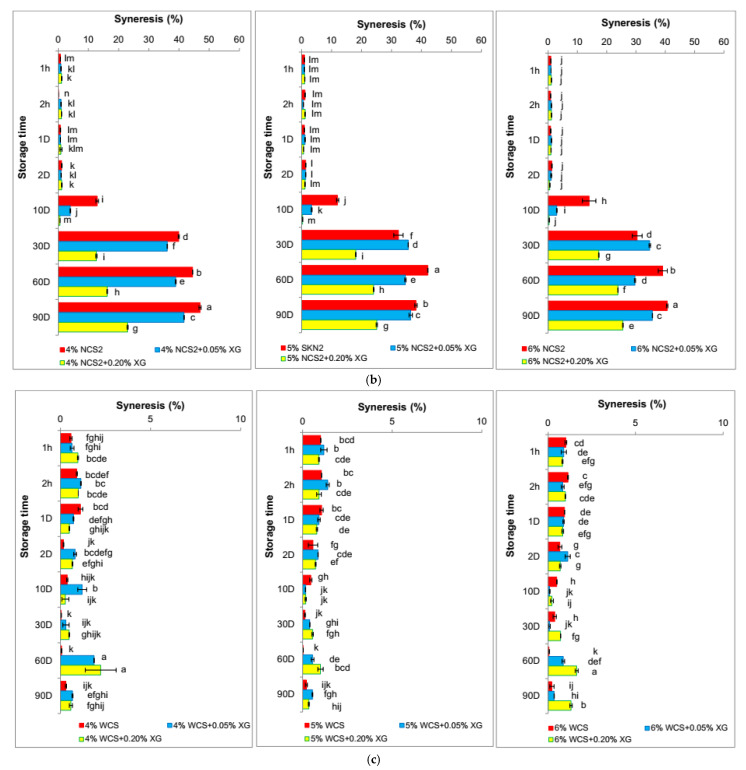
Syneresis of (**a**) 4–6% NCS1 gels without and with 0.05% and 0.2% XG during storage, (**b**) 4–6% NCS2 gels without and with 0.05% and 0.2% XG during storage, (**c**) 4–6% WCS gels without and with 0.05% and 0.2% XG during storage. NCS1—normal corn starch with higher amylose content, NCS2—normal corn starch with lower amylose content, WCS—waxy corn starch, XG—xanthan gum. Results for NCS1, NCS2 and WCS without XG were published in our former paper Sikora et al., 2018 [17]. Parameters denoted with the same letters do not differ statistically at the level of confidence α = 0.05.

Based on the obtained data (Figure 5a,b), no statistically significant differences were found in the syneresis of native corn starch gels until the 2nd day of storage. The phenomenon of syneresis did not occur until the 10th day of refrigerated storage, and from that moment, it increased rapidly until the 90th day. Comparing the obtained results with the previous ones concerning native potato starch [23], one could state that corn starch gels showed structural changes in a shorter time. This was also confirmed by textural (Figure 5a,b) and rheological analysis (Figure 1 and Figure 2). The phenomenon of syneresis was related to the retrogradation of starch, as previous studies have shown that syneresis positively correlates with the content of amylose in gel-forming starches [23]. It was clearly observed in the case of waxy starch gels (Figure 5c). The amount of water separated from waxy maize starch gels was more than 10 times lower than in the case of native maize starch gels. Moreover, the syneresis values of the waxy starch gels change with the storage time. The longer the time, the less syneresis. The presence of the hydrocolloid intensified the phenomenon of syneresis, adversely affecting the amount of water released.

### 3.5. Possible Explanation for Starch–Gum Interaction during Retrogradation Process

The obtained results lead to the following observations. In the presence of xanthan gum, only the short-time retrogradation of starch was reduced. According to Gallant et al. [39], starch granules have a radial “channel” composed of semicrystalline or amorphous components. Langton and Hermansson [40] called them equatorial grooves. Right through these channels, during starch pasting, the amylose flows out of the granules. Other authors also confirm this assumption [41]. As soon as xanthan gum appears in the solution, the outflow of amylose is strongly inhibited. Xanthan surrounds the starch granules with a film and causes deformation of the granules surface, and thus the above-described channels, thereby blocking the leaking of amylose. As a consequence, the rigidity of granules increases, and they now cannot act as fillers and reinforce the continuous phase [20]. Earlier studies confirmed that xanthan gum reduces swelling power and the solubility of corn starch [6,42]. Thus, the observed increase in the viscosity of the two-component system (NCS + XG) compared to starch pastes, alone (Table 1, Table 2 and Table 3), is due to the thickening effect of xanthan gum, which occurs in the continuous phase [42]. This is reflected in the starch retrogradation process, as XG acts as a granule’s stabilizer (less granule disintegration), a fact that was also observed by Fu and BeMiller [6], Hong et al. [28] and He et al. [29].

The way in which xanthan gum interacts with maize starch is a complex phenomenon. Various suggestions appear, but the most probable thesis was presented by Kim et al. [35]. According to the authors, one of the mechanisms in this type of systems is phase separation and association between hydrocolloids and starch molecules. Phase separation has long been a known phenomenon, especially in biopolymer mixtures [35]. According to Achayuthakan and Suphantharika [34], it manifested by limiting the miscibility of components in aqueous solutions and under conditions that promote macromolecular demixing. In the starch–xanthan gum system, mutual exclusion of amylose and hydrocolloid appeared, and as a result, their concentration increases in their microdomains, thus contributing to significant improvement in the viscosity of systems. Most often, this phenomenon is due to thermodynamic incompatibility between two polymers rather than synergistic interactions. Eidam et al. [32] confirmed these assumptions, as well. The presence of xanthan gum, or more specifically its thickening effect, contributes to an increase in the viscosity of the entire system. Mobility of the amylose molecules is restricted; consequently, local association of the helical components is favorable, whereby a three-dimensional network structure stabilized by junction zones is formed. Between these two mechanisms (phase separation and intergranular association) exists competition. The domination of one or the other polysaccharide depends on many factors, including starch–xanthan combination, concentration of polysaccharides [35], and the methods of paste and gels preparation and method of measurements of their properties [6].

## 4. Conclusions

The study confirmed that the process of starch retrogradation consists of two stages. In the first stage, which lasts 48 h, the amylose recrystallization takes place. In the next stage, changes caused by amylopectin retrogradation occurred. Xanthan gum controls short-term structural changes in corn starch through two possible mechanisms—phase separation and intergranular association. The speed of these changes depends on many factors but is mainly related to the amylose content in starch and the amount of hydrocolloid added to the mixture. Mechanical and textural measurement, syneresis studies confirmed destabilization of the starch–hydrocolloid mixture after about 30 days of storage.

The achieved knowledge can be used in the design of long-term foods, such as sauces, dressings, mayonnaise and food concentrates. Thanks to the use of xanthan gum, unfavorable changes in the structure of products and the syneresis can be limited. However, the effectiveness of these action will depend on the gum/starch ratio and the type of product in which this binary system will be applied.

## Figures and Tables

**Table 1:** Parameters of pasting characteristics of NCS1 without and with 0.05% and 0.20% XG addition.

Concentration of Starch (%)	Concentration of XG (%)	T_0_ (°C)	η_max_ (BU)	η_96°C_ (BU)	Setback (BU)
4	0	81.4 ± 1.3 ^a^	88 ± 3 ^h^	88 ± 3 ^g^	54 ± 1 ^f^
	0.05	67.2 ± 2.4 ^c^	122 ± 5 ^g^	122 ± 5 ^f^	60 ± 3 ^e^
	0.20	60.1 ± 0.3 ^d^	157 ± 11 ^f^	143 ± 13 ^e^	41 ± 4 ^g^
5	0	80.1 ± 0.0 ^a^	158 ± 2 ^f^	152 ± 4 ^e^	107 ± 4 ^c^
	0.05	66.7 ± 0.5 ^c^	199 ± 1 ^e^	199 ± 2 ^d^	96 ± 1 ^c,d^
	0.20	65.0 ± 1.3 ^c^	228 ± 5 ^d^	224 ± 3 ^c^	90 ± 1 ^d^
6	0	75.1 ± 0.2 ^b^	256 ± 3 ^c^	236 ± 1 ^c^	175 ± 1 ^a^
	0.05	65.0 ± 0.8 ^c^	297 ± 11 ^b^	282 ± 5 ^b^	152 ± 0 ^b^
	0.20	59.9 ± 1.6 ^d^	353 ± 11 ^a^	348 ± 5 ^a^	158 ± 6 ^b^

T_0_—temperature at the beginning of pasting, η_max_—maximum viscosity, η_96°C_—viscosity at 96 °C, Setback—difference of viscosity between viscosity after cooling the paste to 50 °C and the minimum viscosity, BU—Brabender Units. NC1—normal corn starch with higher amylose content, NC2—normal corn starch with lower amylose content, WCS—waxy corn starch, XG—xanthan gum. Parameters in columns denoted with the same letters do not differ statistically at the level of confidence α = 0.05. Results for NCS1, NCS2 and WCS without XG were published in our former paper Sikora et al., 2018 [17].

**Table 2:** Parameters of pasting characteristics of NCS2 without and with 0.05% and 0.20% XG addition.

Concentration of Starch (%)	Concentration of XG (%)	T_0_ (°C)	η_max_ (BU)	η_96°C_ (BU)	Setback (BU)
4	0	84.4 ± 0.3 ^a^	97 ± 1 ^i^	96 ± 1 ^i^	65 ± 2 ^f^
	0.05	68.1 ± 0.6 ^d^	118 ± 4 ^h^	113 ± 1 ^h^	62 ± 3 ^g^
	0.20	66.8 ± 0.3 ^d,e^	160 ± 4 ^g^	132 ± 1 ^g^	49 ± 4 ^h^
5	0	80.3 ± 1.3 ^b^	179 ± 2 ^f^	167 ± 3 ^f^	127 ± 0 ^c^
	0.05	65.0 ± 0.8 ^e^	211 ± 1 ^e^	209 ± 0 ^e^	103 ± 3 ^d^
	0.20	59.0 ± 0.8 ^g^	260 ± 6 ^d^	256 ± 8 ^d^	94 ± 0 ^e^
6	0	75.2 ± 1.6 ^c^	296 ± ^c^	269 ± 1 ^c^	187 ± 1 ^a^
	0.05	65.9 ± 1.1 ^e^	334 ± 3 ^b^	315 ± 4 ^b^	178 ± 1 ^b^
	0.20	61.4 ± 0.6 ^f^	376 ± 8 ^a^	371 ± 10 ^a^	172 ± 6 ^b^

T_0_—temperature at the beginning of pasting, η_max_—maximum viscosity, η_96°C_—viscosity at 96 °C, Setback—difference of viscosity between viscosity after cooling the paste to 50 °C and the minimum viscosity, BU—Brabender Units. NC1—normal corn starch with higher amylose content, NC2—normal corn starch with lower amylose content, WCS—waxy corn starch, XG—xanthan gum. Parameters in columns denoted with the same letters do not differ statistically at the level of confidence α = 0.05. Results for NCS1, NCS2 and WCS without XG were published in our former paper Sikora et al., 2018 [17].

**Table 3:** Parameters of pasting characteristics of WCS without and with 0.05% and 0.20% XG addition.

Concentration of Starch (%)	Concentration of XG (%)	T_0_ (°C)	η_max_ (BU)	η_96°C_ (BU)	Setback (BU)
4	0	67.9 ± 0.3 ^a^	283 ± 3 ^f^	176 ± 4 ^h^	51 ± 1 ^e^
	0.05	64.2 ± 0.8 ^b^	265 ± 1 ^f^	203 ± 4 ^g^	43 ± 1 ^f^
	0.20	58.6 ± 0.3 ^d^	338 ± 7 ^e^	265 ± 1 ^e^	52 ± 1 ^e^
5	0	67.0 ± 0.0 ^a^	469 ± 4 ^c,d^	244 ± 3 ^f^	78 ± 1 ^d^
	0.05	64.0 ± 0.6 ^b^	447 ± 11 ^d^	278 ± 6 ^d^	77 ± 2 ^d^
	0.20	62.6 ± 1.1 ^c^	487 ± 25 ^c^	323 ± 2 ^c^	85 ± 3 ^c^
6	0	68.0 ± 0.8 ^a^	667 ± 1 ^a^	326 ± 1 ^c^	120 ± 2 ^a^
	0.05	62.5 ± 0.6 ^c^	625 ± 6 ^b^	352 ± 9 ^b^	118 ± 2 ^a,b^
	0.20	61.2 ± 0.3 ^c^	661 ± 7 ^a^	399 ± 8 ^a^	112 ± 4 ^b^

T_0_—temperature at the beginning of pasting, η_max_—maximum viscosity, η_96°C_—viscosity at 96 °C, Setback—difference of viscosity between viscosity after cooling the paste to 50 °C and the minimum viscosity, BU—Brabender Units. NC1—normal corn starch with higher amylose content, NC2—normal corn starch with lower amylose content, WCS—waxy corn starch, XG—xanthan gum. Parameters in columns denoted with the same letters do not differ statistically at the level of confidence α = 0.05. Results for NCS1, NCS2 and WCS without XG were published in our former paper Sikora et al., 2018 [17].

## Data Availability

The data presented in this study are available on request from the corresponding author.
